# Muscarinic M_1_ receptor and cannabinoid CB_1_ receptor do not modulate paraoxon-induced seizures

**DOI:** 10.1002/prp2.100

**Published:** 2014-11-07

**Authors:** Rebecca L Kow, Eugene M Cheng, Kelly Jiang, Joshua H Le, Nephi Stella, Neil M Nathanson

**Affiliations:** Department of Pharmacology, University of WashingtonSeattle, Washington

**Keywords:** Cannabinoid, MAP kinase, muscarinic, organophosphate

## Abstract

One of the major signs of severe organophosphate poisoning is seizures. Previous studies have shown that both muscarinic agonist- and organophosphate-induced seizures require activation of muscarinic acetylcholine receptors in the central nervous system. Seizures induced by the muscarinic agonist pilocarpine require the M_1_ receptor and are modulated by cannabinoid CB_1_ receptors. In this study, we determined whether M_1_ and CB_1_ receptors also regulated seizures induced by the organophosphate paraoxon. We found no differences in seizures induced by paraoxon in wild-type (WT) and M_1_ knockout (KO) mice, indicating that in contrast to pilocarpine seizures, M_1_ receptors are not required for paraoxon seizures. Furthermore, we found that pilocarpine administration resulted in seizure-independent activation of ERK in the hippocampus in a M_1_ receptor-dependent manner, while paraoxon did not induce seizure-independent activation of ERK in the mouse hippocampus. This shows that pilocarpine and paraoxon activated M_1_ receptors in the hippocampus to different extents. There were no differences in seizures induced by paraoxon in WT and CB_1_ KO mice, and neither CB_1_ agonist nor antagonist administration had significant effects on paraoxon seizures, indicating that, in contrast to pilocarpine seizures, paraoxon seizures are not modulated by CB_1_ receptors. These results demonstrate that there are fundamental molecular differences in the regulation of seizures induced by pilocarpine and paraoxon.

## Introduction

The toxic properties of organophosphates make them useful as insecticides but also as weapons for chemical warfare (Newmark 2004; Rusyniak and Nañagas 2004). Organophosphate poisoning is caused by the inhibition of acetylcholinesterase (AChE) due to the formation of a covalent enzyme–inhibitor complex, leading to an increase in acetylcholine (ACh) levels and excessive activation of nicotinic and muscarinic ACh receptors. These receptors mediate the actions of ACh at the neuromuscular junction, target organs of the autonomic nervous system, and neurons of the peripheral and central nervous systems. Signs of organophosphate poisoning include excessive salivation, involuntary movements, respiratory depression, and seizures. Current treatment for organophosphate poisoning typically includes administration of a muscarinic receptor antagonist such as atropine, which blocks many of the autonomic and central nervous system symptoms of organophosphate poisoning, an oxime such as 2-pralidoxime (2-PAM), which reacts with the inactivated AChE to restore enzyme activity, and an anticonvulsant such as a benzodiazepine to stop seizures (Newmark 2004; Rusyniak and Nañagas 2004).

While the respiratory depression caused by organophosphate poisoning is the most immediate life-threatening event, organophosphate-induced seizures can cause massive brain damage that results in long-term neurological impairments (see review by Chen 2012). Muscarinic receptors in the central nervous system mediate the initiation of organophosphate seizures; seizures are blocked by pretreatment with centrally acting muscarinic antagonists but not peripheral-selective muscarinic antagonists or nicotinic receptor antagonists (Capacio and Shih 1991; Shih et al. 1991). There are five muscarinic receptor subtypes, all of which are expressed in the brain (Caulfield and Birdsall 1998). Muscarinic receptors in the brain regulate many functions including learning and memory, locomotion, body temperature, and nociception (see reviews by Wess 2004; Eglen 2006; Wess et al. 2007). Muscarinic agonist-induced seizures require M_1_ receptor activity, as the muscarinic agonist pilocarpine cannot induce seizures in M_1_ knockout (KO) mice but it is still able to induce seizures in mice with deletion of the genes encoding any of the other four muscarinic receptor subtypes (Hamilton et al. 1997; Bymaster et al. 2003).

Muscarinic receptor regulation of organophosphate-induced seizures shares some similarities with muscarinic agonist pilocarpine-induced seizures. Muscarinic antagonists can only inhibit pilocarpine-induced seizures if administered before or shortly after pilocarpine administration (Turski et al. 1984; Clifford et al. 1987). Similarly, muscarinic antagonists only block organophosphate-induced seizures if administered within 20–40 min of organophosphate exposure (McDonough and Shih 1993). These similarities, along with pharmacological studies, have led to the suggestion that the initiation of organophosphate seizures also requires M_1_ receptors as reported for pilocarpine seizures (Harrison et al. 2004; Bhattacharjee et al. 2013). However, a requirement for the M_1_ receptor in organophosphate seizures has not been directly tested.

We recently observed that pilocarpine-induced seizures are modulated by cannabinoid CB_1_ receptors; deletion of the CB_1_ receptor gene or administration of CB_1_ receptor antagonists resulted in an increased susceptibility of mice to pilocarpine-induced seizures (Kow et al. 2014). However, while pilocarpine selectively acts at muscarinic receptors, organophosphates not only inhibit AChE, increasing ACh availability at both muscarinic and nicotinic receptors, but they also inhibit multiple serine hydrolases, raising the possibility that these compounds might exert effects through a different molecular mechanism (Casida and Quistad 2005). These include fatty acid amide hydrolase (FAAH) and monoacylglycerol lipase (MAGL), the enzymes that degrade the endogenous cannabinoids (eCBs) anandamide and 2-AG, respectively. In line with these results, organophosphates have been shown to decrease ligand binding at CB_1_ receptors (Casida and Quistad 2005; Nallapaneni et al. 2006) but do not appear to directly bind to CB_1_ receptors. These results suggest that the reduction in available CB_1_ receptor binding sites is due to increased bioavailability of eCBs to CB_1_ receptors (Casida et al. 2008; Nomura et al. 2008).

This arm of eCB signaling can be enhanced through the activation of M_1_ and M_3_ receptors, which are known to increase eCB release in multiple regions of the brain via activation of phospholipase C *β* (Ohno-Shosaku et al. 2003; Fukudome et al. 2004; Hashimotodani et al. 2005). Thus, organophosphates could increase eCB levels via two different mechanisms: by directly inhibiting the degradation of eCBs by FAAH and MAGL and by directly inhibiting ACh degradation and enhancing M_1_ and M_3_ receptor-mediated eCB production.

In this study, we investigated whether seizures induced by the organophosphate paraoxon require M_1_ receptor activation and are regulated by CB_1_ receptors, thus testing two of the molecular steps involved in seizures induced by paraoxon.

## Materials and Methods

### Animals

M_1_ KO mice were generated and bred at the University of Washington (Hamilton et al. 1997), and backcrossed >12 generations on a C57/Bl6 background. CB_1_ KO mice were obtained from Giovanni Marsicano (Marsicano et al. 2002) and were bred at the University of Washington. For CB_1_ agonist and antagonist seizure studies, C57Bl/6 male mice were purchased from Charles River (Wilmington, MA) and used at 12–13 weeks of age. For studies looking at extracellular signal regulated kinase (ERK) activation in the hippocampus, WT and M_1_ KO male mice were used at 10 weeks of age. All procedures involving animals were approved by the University of Washington Institutional Animal Care and Use Committee.

### Drugs

Paraoxon was purchased from Chem Service (West Chester, PA). Pilocarpine hydrochloride and pyridine-2-aldoxime methochloride (2-PAM) were purchased from Sigma Aldrich (St. Louis, MO). Diazepam (Hospira, Lake Forest, IL) was purchased as a stock solution dissolved in 0.9% saline from the University of Washington Medical Center Pharmacy. SR141716 was obtained from the NIDA Drug Supply Program (Bethesda, MD) and was prepared in pharmasolve/cremophor RH40 (pharmasolve: cremophor RH40: drug, 1:9:40). CP55940 was obtained from the NIDA Drug Supply Program and was prepared in a vehicle solution consisting of cremophor RH40: ethanol: saline (1:1:18). All drugs except for SR141716 and CP55940 were made as stock solutions in 0.9% saline.

### Drug treatments

For seizure studies, male mice were given 90 mg/kg 2-PAM by intraperitoneal (IP) injection 5 min prior to IP injection with paraoxon. Seizure activity was observed for 1 h and scored on a 8-point scale as follows: 0 – no visible response; 1 – sedation, loss of locomotion; 2 – Straub tail, shortened gait; 3 – circling, head bobbing, and/or mouth gaping; 4 – tremors, wild running, and/or cornering; 5 – single myoclonic jerks; 6 – clonic convulsions; 7 – clonic/tonic seizures; 8 – clonic hind limb extension or death. Scoring was done blind to drug treatment and genotype.

For studies in which seizures were prevented, mice were administered 4 mg/kg diazepam by IP injection 15 min prior to IP injection of either 350 mg/kg pilocarpine or 6 mg/kg paraoxon, or equivalent volumes of 0.9% saline for controls. In paraoxon experiments, 2-PAM was also given to mice by IP injection 5 min prior to paraoxon to minimize peripheral toxicity. Fifteen minutes after pilocarpine or paraoxon injection, mice were euthanized by cervical dislocation.

### Tissue processing

Euthanized mice were perfused with 4% paraformaldehyde and their brains removed. Brains were fixed overnight at 4°C in 4% paraformaldehyde in 0.1 mol/L phosphate buffer, pH 7.4. Brains were then soaked in 30% sucrose in PBS before frozen on dry ice. Frozen brains were sectioned at 40 *μ*m, and sections were stored at −20°C in a cryoprotectant solution (30% ethylene glycol, 30% glycerol, 0.1 mol/L phosphate buffer, pH 7.4).

### Phospho-ERK TSA/NeuN immunofluorescence

Phospho-ERK was detected using tyramide signal amplification (TSA) prior to NeuN immunofluorescence. TSA for phospho-ERK was performed as described by Sindreu et al. (2007) using the TSA Cyanine 3 kit (Perkin Elmer, Waltham, MA) with some modifications. To block phosphatase activity, 50 mmol/L NaF was added to every solution up through the phospho-ERK antibody incubation. Free-floating sections were washed multiple times with PBS before they were incubated for 15 min in 1% NaBH_4_ in PBS and 20 min in 0.1 mol/L phosphate buffer, pH 7.4, containing 1.5% H_2_O_2_ and 10% ethanol. Sections were then washed in PBST (PBS + 0.2% Triton X-100) before blocking with TNB blocking buffer (0.1 mol/L Tris-HCl, pH 7.5, 0.15 mol/L NaCl, 0.5% blocking reagent). Sections were incubated at 4°C overnight in 1:5000 rabbit anti-phospho-ERK (Cell Signaling, Danvers, MA) in TNB blocking buffer. After primary antibody incubation, sections were washed with PBST before incubation for 1 h at room temperature in 1:100 anti-rabbit IgG HRP (GE Healthcare, Pittsburgh, PA) in TNB blocking buffer. Sections were washed again with PBST before incubated in Cyanine 3 Tyramide working solution (Cyanine 3 Tyramide stock solution diluted 1:66 in amplification reagent) for 10 min at room temperature.

NeuN immunofluorescence was then performed after residual Cyanine 3 Tyramide solution was removed with multiple PBST washes. Sections were blocked for 1 h at room temperature in blocking solution (0.1 mol/L glycine, 2% bovine serum albumin, 0.05% sodium azide, 10% donkey serum in PBST) before overnight incubation at 4°C in 1:1000 mouse anti-NeuN (Millipore, Billerica, MA) in blocking solution. After multiple washes with PBST, sections were incubated for 3 h at room temperature in 1:500 donkey anti-mouse IgG Alexa Fluor 488 (Invitrogen, Grand Island, NY) in blocking solution. Sections were counterstained with 10 *μ*mol/L Hoechst 33342 in PBS before mounted with Vectashield (Vector Laboratories, Burlingame, CA).

### Quantification of phospho-ERK Fluorescence

Images of hippocampal tissue were taken with a 10X objective on a Nikon (Melville, NY) Eclipse S600 equipped with a QImagine QIClick camera at 8-bit resolution. Hoechst staining and NeuN immunofluorescence were used in order to determine the location and size of the stratum lucidum of each tissue section. Phospho-ERK fluorescence was determined by measuring the mean gray value of the stratum lucidum using ImageJ (NIH, Bethesda, MA). Average background fluorescence was subtracted and the corrected fluorescence values for each tissue section were averaged per animal. Imaging of the tissue and the measurement of phospho-ERK fluorescence were done blind to treatment.

### Data analysis

Seizure severity scores are presented as medians ± upper and lower quartiles. The Mann–Whitney *U*-test was used to test the significance of seizure severity scores. The Fisher’s exact test was used for fractions of mice experiencing a least one clonic–tonic seizure (i.e., seizure severity score ≥7). Immunofluorescence data are presented as means ± SEM. Two-way analysis of variance (ANOVA) was used to determine if there was a genotype effect on the observed phospho-ERK signal following drug or vehicle treatment. Bonferroni-corrected Student *t*-tests were performed between vehicle- and drug-treated mice of the same genotype. *P* values of less than 0.05 were considered statistically significant.

## Results

### M_1_ receptor activity is not necessary for paraoxon-induced seizures

**Figure 1:**
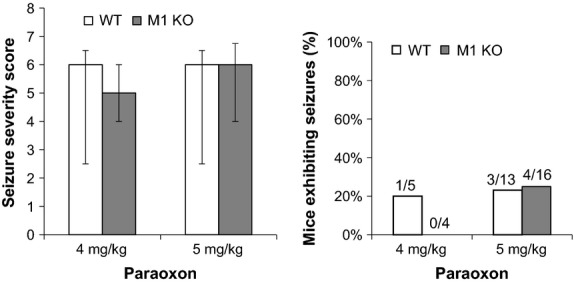
Paraoxon induces seizures to a similar degree in WT and M_1_ KO mice. Seizure severity scores and the proportion of mice having at least one clonic–tonic seizure after 5 mg/kg paraoxon administration was compared in male WT (*n* = 13) and M_1_ KO mice (*n* = 16). Data are presented as medians ± upper and lower quartiles.

Previous work showed that pilocarpine-induced seizure behaviors were reduced and clonic–tonic seizures were absent only from M_1_ KO mice and not from mice lacking any of the four other muscarinic subtypes (Hamilton et al. 1997; Bymaster et al. 2003). Because organophosphates also require muscarinic receptor activity in order to initiate seizures (Capacio and Shih 1991; Shih et al. 1991), organophosphates and muscarinic agonists could share a similar muscarinic receptor requirement for seizure induction. To determine if M_1_ receptor activity was also necessary for paraoxon-induced seizures, we compared seizures induced by 4 and 5 mg/kg paraoxon in WT and M_1_ KO mice. We observed no differences in seizure severity scores or the proportion of mice exhibiting clonic–tonic seizures after 4 or 5 mg/kg paraoxon administration in WT and M_1_ KO mice (Fig. 1). Thus, in contrast to pilocarpine-induced seizures, the M_1_ receptor is not only unnecessary for paraoxon-induced seizures but it also does not significantly modulate sensitivity to paraoxon.

### Pilocarpine, but not paraoxon, activates ERK in a seizure-independent manner in the hippocampus

To further investigate the activity of the M_1_ receptor following pilocarpine or paraoxon administration, we compared the ability of pilocarpine and paraoxon to activate ERK in the hippocampus. The M_1_ receptor is the predominant muscarinic receptor subtype in the hippocampus and cortex (Oki et al. 2005), and M_1_ receptors mediate muscarinic agonist-induced ERK activation in these regions in vitro (Berkeley et al. 2001; Hamilton and Nathanson 2001). We examined the hippocampus because administration of pilocarpine or the organophosphate soman increased hippocampal ERK activation within 15 min in vivo (Berkeley et al. 2002; RamaRao et al. 2011).

**Figure 2:**
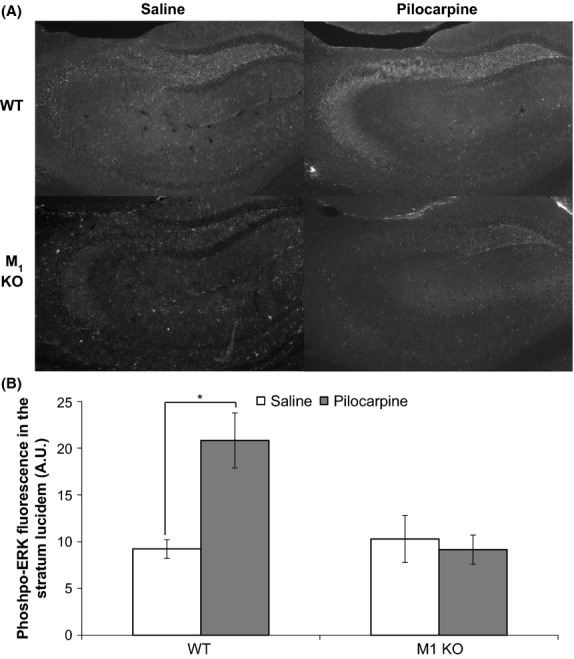
Seizure-independent ERK activation by pilocarpine is absent from M_1_ KO mice. (A) Representative images of phospho-ERK immunofluorescence in the CA3 region of seizure-blocked male WT and M_1_ KO mice 15 min after saline or 350 mg/kg pilocarpine administration. 4 mg/kg diazepam was given 15 min prior to pilocarpine to prevent seizure activity. (B) Quantification of phospho-ERK fluorescence in the stratum lucidum of seizure-blocked male WT and M_1_ KO mice 15 min after saline (*n* = 7 for WT; *n* = 6 for M_1_ KO) or 350 mg/kg pilocarpine (*n* = 8 for WT; *n* = 7 for M_1_ KO) administration. *P < 0.05. Data are presented as means ± SEM.

To distinguish between direct muscarinic receptor-dependent activation of ERK following pilocarpine or paraoxon from ERK activation resulting from seizures, we pretreated mice with diazepam in order to block seizure activity while preserving M_1_ receptor activation. Previous work by Berkeley et al. (2002) demonstrated that pilocarpine could induce ERK activation in the hippocampus even with diazepam pretreatment, indicating that pilocarpine could increase ERK activation in the absence of seizures. In order to confirm that seizure-independent activation of ERK by pilocarpine was M_1_-dependent in vivo, we compared the magnitude and location of seizure-independent ERK activation in the hippocampus of WT and M_1_ KO mice following saline or 350 mg/kg pilocarpine treatment. We found that basal phospho-ERK immunoreactivity in the stratum lucidum was enhanced by pilocarpine administration in WT mice (Fig. 2). While Berkeley et al. (2002) reported an increase in phospho-ERK immunoreactivity in the dentate gyrus and in the CA1 region after pilocarpine administration in mice not pretreated with diazepam, we did not observe an increase in these regions in diazepam-pretreated mice. This suggests that the activation of ERK in the dentate gyrus and CA1 reported by Berkeley et al. (2002) was due to pilocarpine-induced seizure activity. Pilocarpine did not increase phospho-ERK immunoreactivity in the stratum lucidum when administered to M_1_ KO mice, indicating that ERK activation following pilocarpine administration is mediated by the M_1_ receptor.

**Figure 3:**
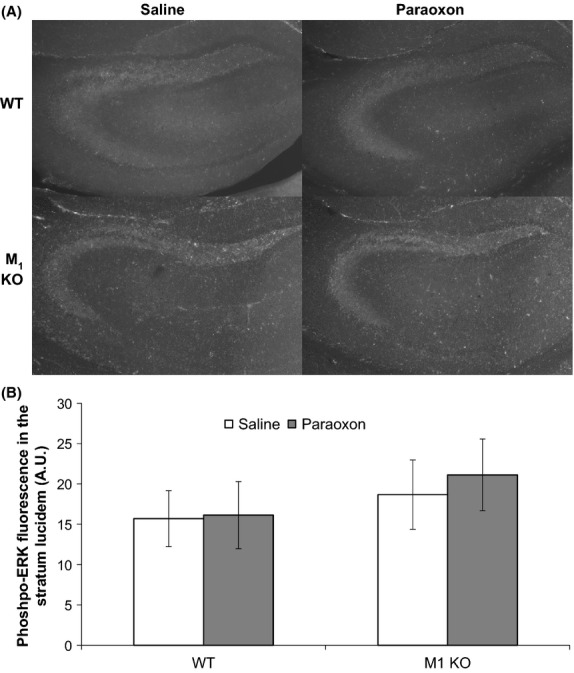
ERK is not activated by paraoxon in seizure-blocked mice. (A) Representative images of phospho-ERK immunofluorescence in the CA3 region of seizure-blocked male WT and M_1_ KO mice 15 min after saline or 6 mg/kg paraoxon administration. 4 mg/kg diazepam was given 15 min prior to paraoxon to prevent seizure activity and 90 mg/kg 2-PAM was given 5 min prior to reduce the effects of acetylcholinesterase inhibition in the periphery. (B) Quantification of phospho-ERK fluorescence in the stratum lucidum of seizure-blocked male WT and M_1_ KO mice 15 min after saline (*n* = 7 for WT; *n* = 6 for M_1_ KO) or 6 mg/kg paraoxon (*n* = 6 for WT; *n* = 7 for M_1_ KO) administration. Data are presented as means ± SEM.

We then compared seizure-independent phospho-ERK levels in the hippocampus of WT and M_1_ KO mice following saline or 6 mg/kg paraoxon treatment. In order to eliminate death due to peripheral organophosphate-induced toxicity, we also administered 2-PAM prior to saline or 6 mg/kg paraoxon. In contrast to pilocarpine, paraoxon did not increase phospho-ERK immunoreactivity in the hippocampus of either WT or M_1_ KO mice (Fig. 3). The inability of paraoxon to increase ERK activation in a seizure-independent manner provides strong evidence that paraoxon administration produces less activation of M_1_ receptors in the hippocampus than pilocarpine administration.

### CB_1_ receptor activity does not affect paraoxon-induced seizures

Recently, we observed that pilocarpine-induced seizures are modulated by CB_1_ receptor activity (Kow et al. 2014). Loss of CB_1_ receptor activity either through pharmacological antagonism or genetic deletion increased the seizure severity scores seen with submaximal doses of pilocarpine, suggesting that release of eCBs likely modulates seizure induction due to the administration of pilocarpine. Loss of CB_1_ receptor activity also increased the severity of kainic acid and spontaneous seizures, indicating that eCB activity at CB_1_ receptors was generally anticonvulsive (Marsicano et al. 2003; Wallace et al. 2003). Organophosphates can inhibit FAAH and MAGL, causing significant increases in eCB levels (Casida et al. 2008; Nomura et al. 2008). Furthermore, 0.4 mg/kg paraoxon significantly inhibited FAAH activity in vivo and caused greater toxicity in rats with reduced CB_1_ receptor expression, suggesting that eCBs can modulate paraoxon toxicity via CB_1_ activity (Nallapaneni et al. 2006).

**Figure 4:**
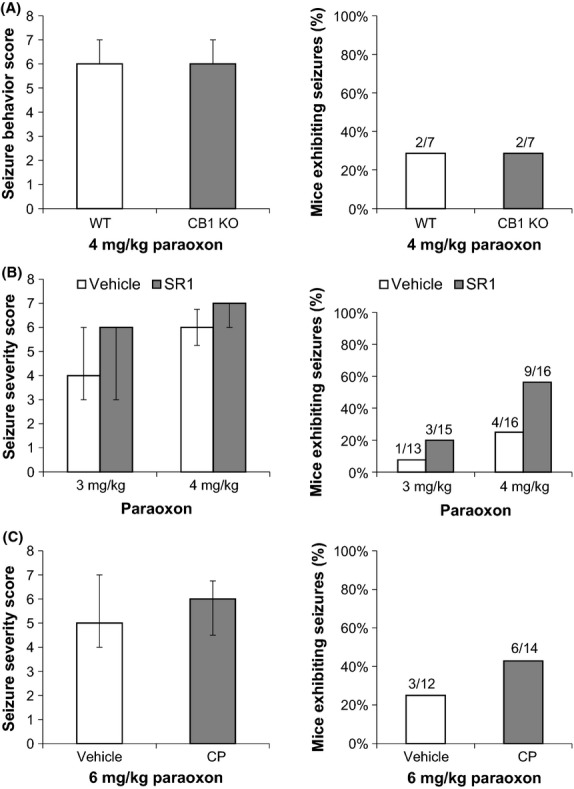
CB_1_ activity does not alter severity of paraoxon seizures. Seizure severity scores and the proportion of mice having at least one clonic–tonic seizure were compared between the following groups of mice. (A) Male CB_1_ KO (*n* = 7) and WT (*n* = 7) littermates. (B) CB_1_ receptor antagonist and vehicle-pretreated mice. SR141716 (SR1, 10 mg/kg) or the corresponding vehicle was given 2 h prior to 3 mg/kg paraoxon (*n* = 13 for vehicle; *n* = 15 for SR1) or 4 mg/kg paraoxon (*n* = 16 for vehicle; *n* = 16 for SR1). (C) CB_1_ receptor agonist or vehicle. CP 55940 (CP, 0.3 mg/kg) or the corresponding vehicle was given 30 min prior to 6 mg/kg paraoxon (*n* = 12 for vehicle; *n* = 14 for CP). Data are presented as medians ± upper and lower quartiles.

Based on this evidence, we sought here to determine whether loss of CB_1_ receptor activity affected paraoxon-induced seizures by comparing seizure activity induced by 4 mg/kg paraoxon in WT and CB_1_ KO mice. We saw no differences in seizure severity scores or proportion of mice experiencing clonic–tonic seizures between WT and CB_1_ KO mice (Fig. 4A). Consistent with this result, pretreatment with the CB_1_ receptor antagonist SR141716 (SR1) also did not significantly alter either seizure severity scores or the proportion of mice experiencing clonic–tonic seizures after 3 or 4 mg/kg paraoxon treatment (Fig. 4B). These results show that paraoxon-induced seizures, in contrast to pilocarpine-induced seizures, are unaffected by the lack of CB_1_ receptor activity.

Administration of 0.4 mg/kg paraoxon was reported to cause significant inhibition of FAAH, which should result in increased levels of eCBs; nevertheless, CB_1_ agonists were still able to reduce paraoxon-induced increases in involuntary movements and parasympathomimetic toxicity (Nallapaneni et al. 2006). To determine whether CB_1_ agonist treatment could similarly reduce the severity of paraoxon-induced seizures, we compared seizures induced by paraoxon in vehicle- and CB_1_ agonist CP55940 (CP)-pretreated mice. CP pretreatment did not alter seizure scores or the proportion of mice that experienced clonic–tonic seizures (Fig. 4C). Altogether these results suggest that paraoxon-induced seizures are unaffected by CB_1_ receptor activity.

## Discussion

Since the identification of the M_1_ receptor as the muscarinic receptor subtype necessary for pilocarpine-induced seizures (Hamilton et al. 1997), several laboratories have suggested that blockade of M_1_ receptors in the central nervous system would be sufficient in preventing organophosphate-induced seizures (Sheffler et al. 2009; Bhattacharjee et al. 2013). Pharmacological studies performed in slice preparations provided initial evidence that blocking M_1_ receptor activity would prevent organophosphate-induced seizure activity (Harrison et al. 2004). However, even though organophosphates initiate seizures in a muscarinic receptor-dependent manner (Capacio and Shih 1991; Shih et al. 1991), we observed that paraoxon-induced seizures do not require M_1_ receptor activity.

Paraoxon-induced seizures were not only present but were remarkably similar in severity when measured in WT and M_1_ KO mice. While this finding does not exclude the M_1_ receptor from possibly contributing to the initiation of paraoxon-induced seizures in WT mice, it does suggest that other muscarinic receptor subtypes can fully initiate paraoxon seizures in the absence of the M_1_ receptor. A difference in the brain regions involved in seizure initiation could explain the differential M_1_ receptor requirement for pilocarpine- and paraoxon-induced seizures. Pilocarpine caused an increase in high-voltage spiking in the hippocampus followed by the cortex, suggesting that pilocarpine-induced seizures begin in the hippocampus (Turski et al. 1984). In contrast, the organophosphate soman did not display a consistent sequence of activation; sometimes it increased hyperexcitability in the cortex before the hippocampus and sometimes after the hippocampus (McDonough and Shih 1993). In addition, ACh could evoke seizure activity when stereotaxically injected in the amygdala, hippocampus, or cortex, although the most sensitive region was the “area tempestus” (Gale 1988). A more detailed analysis of excitability in the brain following paraoxon administration could determine which regions are most sensitive to paraoxon-induced excitability.

The difference in seizure-independent ERK activation following pilocarpine and paraoxon is consistent with the different requirements for the M_1_ muscarinic receptor in seizure induction. Using doses of pilocarpine and paraoxon that would normally induce significant increases in seizure severity scores in a majority of mice, we observed that only pilocarpine administration caused a seizure-independent increase in ERK activation. The fact that the pilocarpine-induced increase in ERK activation was absent from M_1_ KO mice confirmed that the M_1_ receptor mediated muscarinic agonist-induced ERK activation in the hippocampus as seen previously with carbachol (Berkeley et al. 2001). The concentrations of agonist required for M_1_-mediated activation of MAPK are relatively high (Berkeley et al. 2001; Hamilton and Nathanson 2001), so it is likely that there is little if any spare receptor reserve for this response. If we use ERK activation as a measure of M_1_ receptor activity in the hippocampus, then the lack of ERK activation following paraoxon administration in WT mice suggests that paraoxon administration does not increase M_1_ receptor activity to a sufficient extent in the hippocampus to lead to ERK activation.

Even though ERK activation is not necessary for seizure induction, increased ERK phosphorylation in the hippocampus occurs in response to spontaneous seizures, pilocarpine-induced seizures, and soman-induced seizures (Berkeley et al. 2002; Houser et al. 2008; RamaRao et al. 2011). This implies that paraoxon does not sufficiently stimulate M_1_ receptors in the hippocampus to induce ERK activation, and that paraoxon seizure-induced ERK activation is mediated by other receptors present in the hippocampus which are activated by the many neurotransmitters released during seizures. These include the metabotropic and ionotropic glutamate receptors, *β*-adrenergic receptors, and serotonin receptors, all of which have been shown to activate ERK activity in the hippocampus (Sala et al. 2000; Errico et al. 2001; Berkeley and Levey 2003).

We also observed a difference in CB_1_ receptor regulation of pilocarpine- and paraoxon-induced seizures. Both CB_1_ receptor antagonism and genetic deletion of CB_1_ increased the severity of pilocarpine-induced seizures, indicating that eCB activity at CB_1_ receptors may be responsible for controlling the sensitivity and incidence of pilocarpine-induced seizures (Kow et al. 2014). In sharp contrast, treatment with CB_1_ receptor antagonists or CB_1_ receptor agonists or deletion of the gene encoding the CB_1_ receptor had no effect on the severity of paraoxon-induced seizures, emphasizing a fundamental molecular difference with pilocarpine-induced seizures. Indeed, the lack of CB_1_ receptor modulation of paraoxon-induced seizures is unexpected, considering that behavior’s characteristic of increased cholinergic activity caused by 0.4 and 0.6 mg/kg paraoxon was affected by CB_1_ agonist treatment or decreases in CB_1_ receptor expression (Nallapaneni et al. 2006). Nallapaneni et al. (2006) also observed significant block of FAAH activity and CB_1_ receptor binding sites by paraoxon in vivo*,* indicating that paraoxon interacts with components of the cannabinoid system. However, if CB_1_ receptor activity modulated paraoxon-induced seizures, then at least one of the following should have been observed. If the anticonvulsive activity of CB_1_ receptors was already maximal due to eCB activity, then reduction of CB_1_ receptor activity by CB_1_ antagonist pretreatment should have increased paraoxon-induced seizures severity. On the other hand, if eCB activity at CB_1_ receptors was too low to significantly affect seizure severity, then increasing CB_1_ receptor activity with addition of CB_1_ agonists should have reduced paraoxon-induced seizure severity. The inability of both CB_1_ agonists and CB_1_ antagonists to alter the severity or the proportion of clonic–tonic seizures induced by paraoxon indicates that CB_1_ receptor activity does not regulate paraoxon-induced seizure activity.

While paraoxon-induced seizures were not regulated by CB_1_ receptors, we cannot exclude the possibility that seizures induced by other organophosphates might be sensitive to CB_1_ receptor activity. The ability of CB_1_ receptors to regulate nonseizure toxic effects of organophosphates has been reported to be different depending on the organophosphate examined. While CB_1_ agonists decreased both involuntary movements and salivation caused by paraoxon administration, they only decreased involuntary movements and not salivation following diisopropylfluorophosphate (DFP) administration (Nallapaneni et al. 2006, 2008). Unexpectedly, loss of CB_1_ receptor expression did not increase involuntary movement or salivation caused by chlorpyrifos administration (Baireddy et al. 2011). These results are not inconsistent, since a multitude of factors, including pharmacokinetic properties and ability to inhibit FAAH and/or MAGL, may influence the ability of CB_1_ receptor activity to regulate different aspects of organophosphate toxicity. Further studies are necessary to determine whether modulation of the cannabinoid system, especially the activity of CB_1_ receptors, is a reasonable strategy to treat any of the various organophosphate-induced toxicities including organophosphate-induced seizures.

In summary, we have identified fundamental molecular differences in the roles of the M_1_ and CB_1_ receptors in seizures induced by pilocarpine and paraoxon. Accordingly, drugs targeting the cannabinoid system are unlikely to represent an alternative to current therapies for managing organophosphate seizures. While further studies on the regulation of cholinergic agent-induced seizures by other muscarinic agonists and organophosphates will help determine the shared and the cholinergic agent-specific mechanisms of cholinergic seizure induction, our results suggest that therapies appropriate for the prevention or treatment of pilocarpine-induced seizures may not necessarily be effective in the prevention or treatment of organophosphate-induced seizures.

## Abbreviations

2-PAM: 2-pralidoxime
ACh: acetylcholine
AChE: acetylcholineesterase
CB_1_: cannabinoid receptor 1
CP: CP55940, 2-[(1R,2R,5R)-5-hydroxy-2-(3-hydroxypropyl)cyclohexyl[-5-(2-methyloctan-2-yl)phenol
eCB: endogenous cannabinoid
FAAH: fatty acid amide hydrolase
IP: intraperitoneal
MAGL: monoacylglycerol lipase
SR1: SR141716, 5-(4-Chlorophenyl)-1-(2,4-dichloro-phenyl)-4-methyl-*N*-(piperidin-1-yl)-1*H*-pyrazole-3-carboxamide
TSA: tyramide signal amplification
